# Protective Effect of Procyanidin B2 against CCl_4_-Induced Acute Liver Injury in Mice

**DOI:** 10.3390/molecules200712250

**Published:** 2015-07-03

**Authors:** Bing-Ya Yang, Xiang-Yu Zhang, Sheng-Wen Guan, Zi-Chun Hua

**Affiliations:** State Key Laboratory of Pharmaceutical Biotechnology, School of Life Sciences, Nanjing University, Nanjing 210023, Jiangsu, China; E-Mails: yangbingya2005@163.com (B.-Y.Y.); zhangxiangyu_321@163.com (X.-Y.Z.); guanswnju@163.com (S.-W.G.)

**Keywords:** procyanidin B2, acute liver injury, oxidative stress, inflammatory response, apoptosis

## Abstract

Procyanidin B2 has demonstrated several health benefits and medical properties. However, its protective effects against CCl_4_-induced hepatotoxicity have not been clarified. The present study aimed to investigate the hepatoprotective effects of procyanidin B2 in CCl_4_-treated mice. Our data showed that procyanidin B2 significantly decreased the CCl_4_-induced elevation of serum alanine aminotransferase activities, as well as improved hepatic histopathological abnormalities. Procyanidin B2 also significantly decreased the content of MDA but enhanced the activities of antioxidant enzymes SOD, CAT and GSH-Px. Further research demonstrated that procyanidin B2 decreased the expression of TNF-α, IL-1β, cyclooxygenase-2 (COX-2) and inducible nitric oxide synthase (iNOS), as well as inhibited the translocation of nuclear factor-kappa B (NF-κB) p65 from the cytosol to the nuclear fraction in mouse liver. Moreover, CCl_4_-induced apoptosis in mouse liver was measured by (terminal-deoxynucleotidyl transferase mediated nick end labeling) TUNEL assay and the cleaved caspase-3. Meanwhile, the expression of apoptosis-related proteins Bax and Bcl-xL was analyzed by Western blot. Results showed that procyanidin B2 significantly inhibited CCl_4_-induced hepatocyte apoptosis, markedly suppressed the upregulation of Bax expression and restored the downregulation of Bcl-xL expression. Overall, the findings indicated that procyanidin B2 exhibited a protective effect on CCl_4_-induced hepatic injury by elevating the antioxidative defense potential and consequently suppressing the inflammatory response and apoptosis of liver tissues.

## 1. Introduction

The liver is an important metabolic organ with vital roles in several physiological processes, including proteins synthesis, glucose homeostasis and detoxification, as well as utilization and cycling of various nutrients [1]. Generally, liver injury is considered a result of exposure to high levels of environmental toxins, which are associated with metabolic dysfunctions, ranging from the transient elevation of liver enzymes to life-threatening hepatic fibrosis, liver cirrhosis and even hepatocellular carcinoma [2]. Substantial evidence has implicated oxidative stress and inflammation in the etiology of liver injury [3]. Consequently, CCl_4_, a chemical hepatotoxin, that produces reactive free radicals trichloromethyl radical (CCl_3_) and a proxy trichloromethyl radical (CCl_3_O_2_) when metabolized, has been frequently used to investigate the hepatoprotective effects of drugs and plant extracts as a solvent for induction of hepatic damage in animal models. CCl_4_ increases lipid peroxidation and protein oxidation in hepatic cells, as well as induces liver damage and apoptosis [4].

Grape seed procyanidin extract (GSPE) demonstrates various therapeutic properties, such as radical scavenging, and several health benefits, including anti-ulcer, anti-allergy, anti-dental caries, and antitumor activities [5,6]. However, GSPE is a complex mixture of structurally related components, and the biological properties of individual components have not been explicitly determined. Procyanidin B2, one of the main components of GSPE, had been found to exert various anti-inflammatory and antitumor effects at the same concentrations, which were greater than those of other components of GSPE, such as procyanidins B1, B4, and B5 [7,8,9]. To date, the hepatoprotective effect of procyanidin B2 has not been fully investigated.

Therefore, the present study was designed to examine the protective effects of procyanidin B2 against CCl_4_-induced acute hepatic injury, with particular attention to oxidative stress, inflammatory response and apoptosis.

## 2. Results and Discussion

### 2.1. Procyanidin B2 Protects against CCl_4_-Induced Hepatic Dysfunction

**Figure 1:**
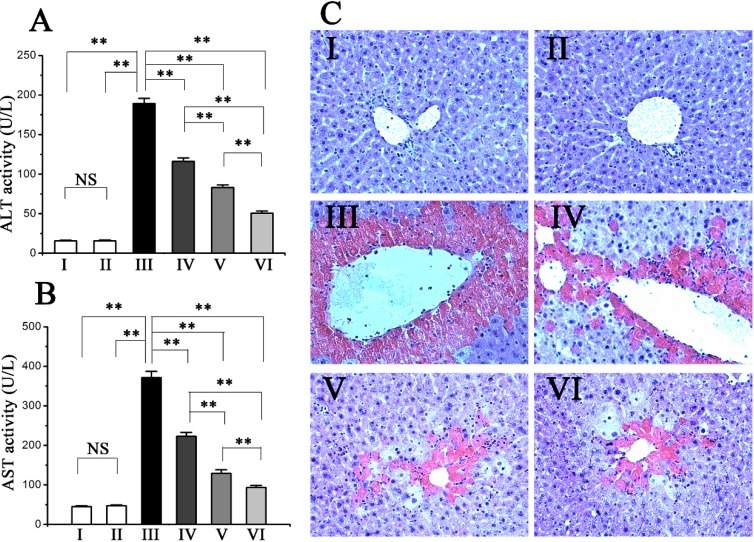
Pretreatment effects of procyanidin B2 on CCl_4_-induced hepatic injury. Serum ALT (**A**); AST (**B**) activities were measured; (**C**) Mouse liver sections stained with H & E to show histopathology of livers (magnification: 200×). Values expressed as mean ± SE in each group. NS: no significant, ** *p* < 0.01, *n* = 10. Animals were divided into following groups: I, normal control; II, procyanidin B2 control; III, model; IV, procyanidin B2 (25 mg∙kg^−1^) + CCl_4_; V, procyanidin B2 (50 mg∙kg^−1^) + CCl_4_; and VI, procyanidin B2 (100 mg∙kg^−1^) + CCl_4_.

Serum activities of alanine aminotransferase (ALT) and aspartate aminotransferase (AST) are biochemical markers of acute hepatic damage. The serum activities of both ALT and AST were significantly increased in the model group compared with those in the normal group (Figure 1A,B). However, pre-administration of procyanidin B2 at three different doses for seven consecutive days significantly prevented the CCl_4_-induced increase of serum activity of ALT and AST. In contrast, administration of procyanidin B2 (100 mg∙kg^−1^) in the procyanidin B2 control group did not alter the level of hepatic markers.

### 2.2. Procyanidin B2 Alleviated CCl_4_-Induced Histopathological Changes in the Liver

Histopathological evaluation of liver sections stained with hematoxylin and eosin (H & E) was performed under a light microscope (Figure 1C). Normal liver architecture with a well-preserved cytoplasm, prominent nucleus and nucleolus, and visible central veins and thin sinusoids were shown in the normal group. In the model group, apparent liver injuries, which were characterized by severe loss of hepatic architecture and condensed nuclei around the central vein, were observed. However, the pre-administration of procyanidin B2 reversed the hepatic lesions. No statistical differences were observed between the procyanidin B2 control group and the normal group.

### 2.3. Procyanidin B2 Suppressed CCl_4_-Induced Oxidative Liver Injury

**Figure 2:**
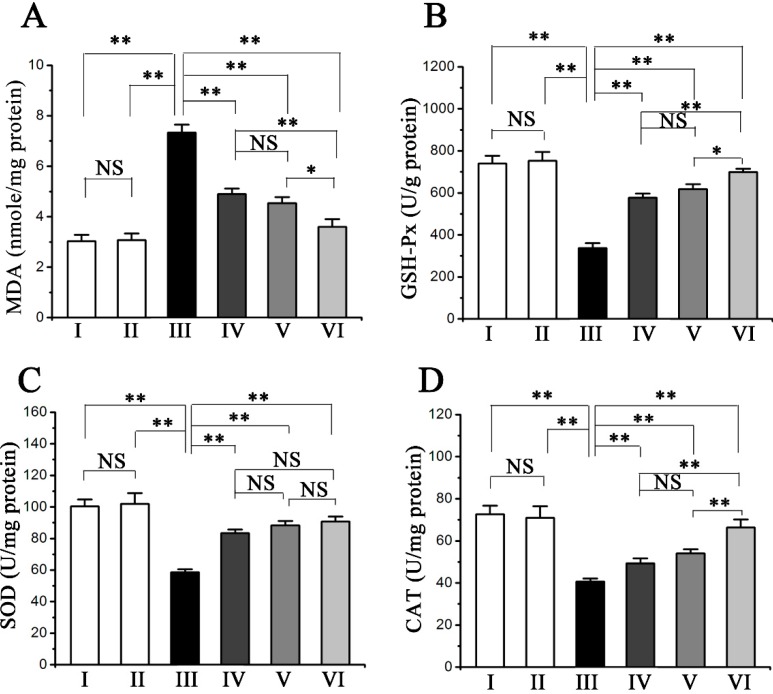
Pretreatment effects of procyanidin B2 on CCl_4_-induced lipid peroxidation production MDA (**A**); and the activities of antioxidant enzymes GSH-Px (**B**); SOD (**C**); and CAT (**D**) in the livers. Animals were divided into following groups: I, normal control; II, procyanidin B2 control; III, model; IV, procyanidin B2 (25 mg∙kg^−1^) + CCl_4_; V, procyanidin B2 (50 mg∙kg^−1^) + CCl_4_; and VI, procyanidin B2 (100 mg∙kg^−1^) + CCl_4_. Values expressed as mean ± SE in each group. NS: no significant, * *p* < 0.05, ** *p* < 0.01, *n* = 7.

The hepatic level of malondialdehyde (MDA) was assessed as an indicator of lipid peroxidation in oxidative liver damage (Figure 2A). CCl_4_ treatment markedly increased the hepatic MDA level compared with the normal group, whereas the pre-administration of procyanidin B2 significantly decreased the MDA levels. The activity of the antioxidant enzymes in the liver was also assessed. The hepatic activities of glutathione peroxidase (GSH-Px), superoxide dismutase (SOD), and catalase (CAT) were conspicuously decreased in CCl_4_-treated mice compared with those in the normal group, whereas the pre-administration of procyanidin B2 significantly reversed the decreased activities of GSH-Px, SOD, and CAT (Figure 2B–D).

### 2.4. Procyanidin B2 Inhibited CCl_4_-Induced Pro-Inflammatory Response

**Figure 3:**
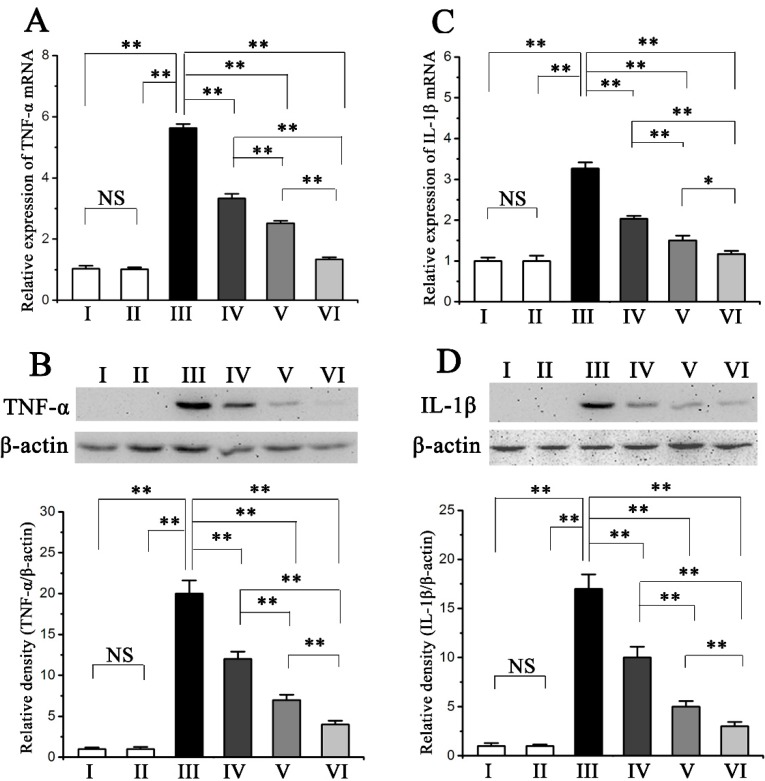
Pretreatment effects of procyanidin B2 on CCl_4_-induced expression of inflammatory cytokine. Quantitative real time PCR was perform to measure TNF-α (**A**) and IL-1β (**C**) mRNA expression levels in response to CCl_4_ and procyanidin B2; Western blot analysis was perform to measure TNF-α (**B**) and IL-1β (**D**) protein expression levels in response to CCl_4_ and procyanidin B2. Values expressed as mean ± SE in each group. NS: no significant, * *p* < 0.05, ** *p* < 0.01, *n* = 7. Animals were divided into following groups: I, normal control; II, procyanidin B2 control; III, model; IV, procyanidin B2 (25 mg∙kg^−1^) + CCl_4_; V, procyanidin B2 (50 mg∙kg^−1^) + CCl_4_; and VI, procyanidin B2 (100 mg∙kg^−1^) + CCl_4_.

CCl_4_ treatment significantly increased the hepatic TNF-α and IL-1β mRNA and protein expression compared with those of the normal group, implying an induction of a severe inflammatory response (Figure 3A–D). However, the pre-administration of procyanidin B2 apparently repressed the mRNA and protein expression of hepatic TNF-α and IL-1β (Figure 3A–D).

**Figure 4:**
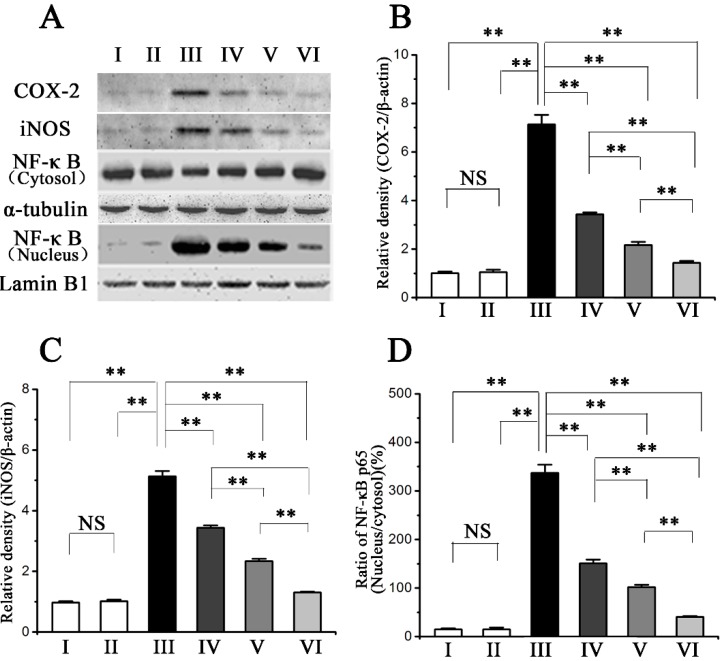
Pretreatment effects of procyanidin B2 on CCl_4_-induced expression of inflammatory mediators. (**A**) Western blot analysis of COX-2, iNOS and NF-κB proteins in response to CCl_4_ and procyanidin B2; (**B**) Relative density analysis of COX-2 protein bands; (**C**) Relative density analysis of iNOS protein bands; (**D**) Relative density of the analysis of the NF-κB p65 protein bands expressed as the ratio in nucleus and cytosol. The normal control is set as 1.0. Values expressed as mean ± SE in each group. NS: no significant, ** *p* < 0.01, *n* = 7. Animals were divided into following groups: I, normal control; II, procyanidin B2 control; III, model; IV, procyanidin B2 (25 mg∙kg^−1^) + CCl_4_; V, procyanidin B2 (50 mg∙kg^−1^) + CCl_4_; and VI, procyanidin B2 (100 mg∙kg^−1^) + CCl_4_.

COX-2 and iNOS are key enzymes to produce inflammatory factors prostaglandin and NO, respectively. Western blot analysis showed the inhibitory effects of procyanidin B2 on hepatic COX-2 and iNOS protein expression. As shown in Figure 4A–C, CCl_4_ treatment significantly upregulated the hepatic COX-2 and iNOS protein levels compared with those in the normal group, which were attenuated by the pre-administration of procyanidin B2.

Accumulated evidence showed that the activation of NF-κB was closely associated with inflammation. To further investigate the molecular mechanism of inflammation in the mouse liver, we measured the translational levels of NF-κB p65. NF-κB p65 expression in the nuclear fractions were significantly increased in the model group compared with normal group (Figure 4A,D). Accordingly, NF-κB p65 levels in the cytoplasmic fractions were significantly reduced in the model group, indicating a translocation of NF-κB p65 and the activation of the correlating signaling pathways. However, the pre-administration of procyanidin B2 markedly inhibited the translocation of NF-κB p65.

### 2.5. Procyanidin B2 Decreases CCl_4_-Induced Apoptosis of Hepatocytes

**Figure 5:**
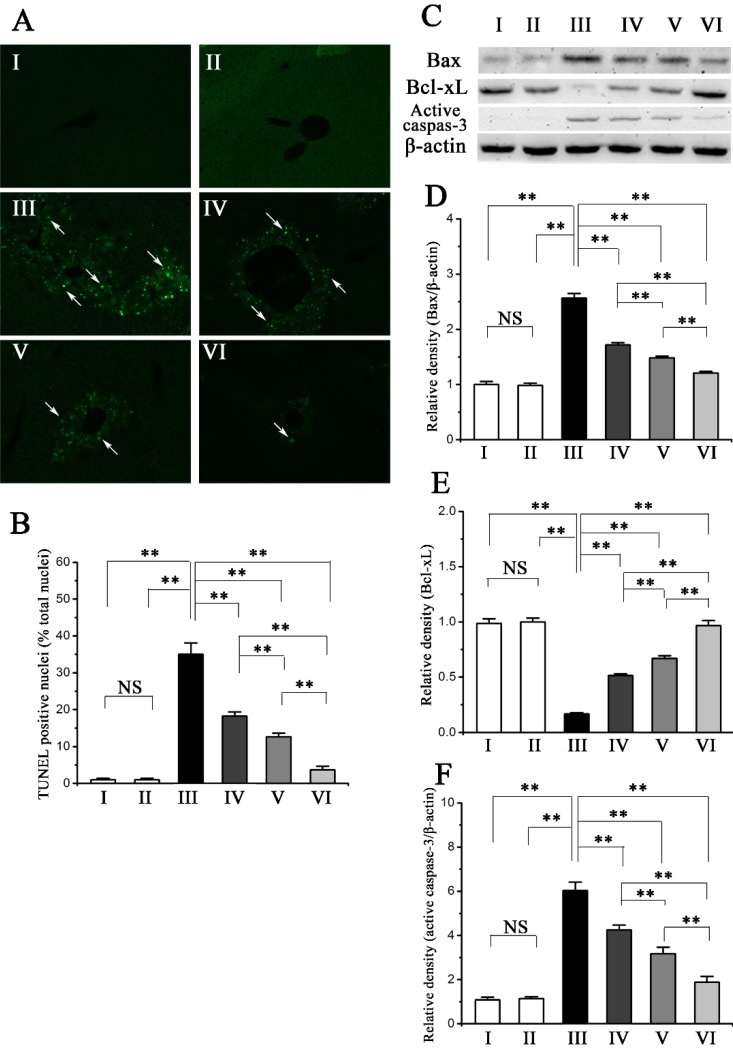
Pretreatment effects of procyanidin B2 on CCl_4_-induced apotosis. (**A**) TUNEL stained liver sections (magnification, 100×), green fluorescence indicated the positive cells (arrows); (**B**) Statistic analysis of the relative proportion of TUNEL positive cells in the liver of mice; (**C**) Western blot analysis of Bax, Bcl-xL and active caspase-3 proteins in response to CCl_4_ and procyanidin B2; (**D**) Relative density analysis of Bax protein bands; (**E**) Relative density analysis of Bcl-xL protein bands; (**F**) Relative density analysis of active caspase-3 protein bands. Values expressed as mean ± SE in each group. NS: no significant, ** *p* < 0.01. *n* = 7. Animals were divided into following groups: I, normal control; II, procyanidin B2 control; III, model; IV, procyanidin B2 (25 mg∙kg^−1^) + CCl_4_; V, procyanidin B2 (50 mg∙kg^−1^) + CCl_4_; and VI, procyanidin B2 (100 mg∙kg^−1^) + CCl_4_.

Previous studies have reported severe hepatocyte apoptosis in CCl_4_-induced acute liver injury [4]. We performed TUNEL staining to assess the protective ability of procyanidin B2 against CCl_4_-induced hepatocytes apoptosis. The number of TUNEL-positive cells in the liver of the model group was significantly increased as compared to the normal group (Figure 5A,B), which was obviously attenuated by the pre-administration of procyanidin B2. The analysis in the activate caspase-3 in hepatocytes also revealed that the pre-administration of procyanidin B2 inhibit the increased apoptosis induced by CCl_4_ treatment (Figure 5C,F).

To determine the mechanism underlying the anti-apoptotic effects of procyanidin B2, the expression of the apoptosis-related genes Bax and Bcl-xL in hepatocytes was detected in Western blot. The expression of Bax, a proapoptotic Bcl-2 family member, increased in the model group compared with that in the normal group, whereas the expression of prosurvival protein Bcl-xL in the model group decreased compared with that in the normal group (Figure 5C–E). However, the pre-administration of procyanidin B2 can significantly downregulate the levels of Bax expression and significantly upregulate the levels of Bcl-xL expression compared with those in the model group.

### 2.6. Discussion

CCl_4_ is a potent hepatotoxic agent that has been extensively used to establish animal models for screening of the hepatoprotective activities of drugs [10]. The current study showed that intraperitoneal injection of CCl_4_ significantly elevated serum ALT and AST activities. AST and ALT are normally localized in both cytoplasm and mitochondria of hepatocytes, whereas increased serum AST and ALT levels suggest the induction of acute hepatotoxicity by CCl_4_. Histopathological examination also reflected the severity of hepatic injury. The obtained results agreed with those of the previous reports [11]. However, pre-administered procyanidin B2 significantly decreased the CCl_4_-induced serum activities of ALT and AST, as well as reduced the CCl_4_-induced histopathological changes in liver. These findings suggest that procyanidin B2 can exert protective activity against CCl_4_-induced liver injury.

Oxidative stress has been accepted as one of the principal causes of CCl_4_-induced hepatic injury, which is mediated by the production of free radical derivatives of CCl_4_ and is responsible for cell membrane damage and the consequent release of marker enzymes of hepatotoxicity [10,12]. Oxidative injury induced by CCl_4_ can be monitored in experimental animals by detecting oxidative stress parameters, such as MDA, SOD, CAT and GSH-Px [13]. MDA is one of lipid peroxidative product, which has been used as a biomarker of lipid peroxidation for several decades [14]. Furthermore, the increase of MDA has been considered a key feature in liver injury [15]. Our investigation revealed that pre-administered procyanidin B2 significantly inhibited the increase of MDA in the liver of the CCl_4_-treated mice. SOD is an effective antioxidant enzyme catalyzing the dismutation of superoxide anions into H_2_O_2_ [16], while CAT is a widely distributed heme-containing enzyme catalyzing the decomposition of excessive H_2_O_2_ [17,18]. GSH-Px is an important enzyme that catalyzes the reduction of H_2_O_2_ and hydroperoxides into non-toxic products and then terminates the chain reaction of lipid peroxidation [19]. Lipid peroxides or reactive oxygen species (ROS) can easily inactivate these antioxidant enzymes [20]. The current results showed that SOD, CAT and GSH-Px activity were significantly decreased in the liver in response to CCl_4_ treatment, thereby indicating increased oxidative damage to the liver. However, SOD, CAT and GSH-Px activities were significantly elevated by the pre-administration of procyanidin B2 to CCl_4_-intoxicated mice, suggesting the ability of procyanidin B2 to restore and maintain the activities of SOD, CAT and GSH-Px in CCl_4_-damaged liver. Therefore, the administration of procyanidin B2 can effectively protect against the CCl_4_-induced hepatic lipid peroxidation via preventing the decrease of activities of GSH-Px, SOD and CAT in mice induced by CCl_4_.

Inflammation is another important pathological mechanism propagating CCl_4_-induced liver injury [21]. Accumulating evidence has revealed that CCl_4_ and excessive ROS induced by CCl_4_ probably activate Kupffer cells, which can mediate the hepatic inflammation process by producing TNF-α, IL-1β, and other pro-inflammatory cytokines [22,23]. Various inflammatory factors have been strongly correlated with the NF-κB pathway in CCl_4_-induced acute liver injury [24]. Many reports have shown that NF-κB leads to the expression of pro-inflammatory cytokines [25,26]. In our research, CCl_4_ treatment significantly upregulated the expression of TNF-α, IL-1β, COX-2 and iNOS, as well as increased the translocation of NF-κB p65 from the cytosol to the nuclear fraction in the mouse liver. However, the pre-administration of procyanidin B2 markedly inhibited the upregulation of these pro-inflammatory cytokines and the translocation of NF-κB p65 to the nucleus. These results suggested that procyanidin B2 can alleviate liver injury caused by CCl_4_ by suppressing the inflammatory response.

Several previous studies have demonstrated that hepatocyte apoptosis can be triggered by various chemical agents, including CCl_4_ [4]. In our study, TUNEL staining indicated that CCl_4_ treatment significantly increased the rate of apoptosis in the model group (Figure 4), which was significantly reduced by pre-administration of procyanidin B2. Apoptosis can be induced by the mitochondrial pathway, the endoplasmic reticulum-mediated pathway, and the death receptor-mediated pathway [27]. The mitochondrial apoptotic pathway has been associated with various toxins and oxidative stress [28]. The mitochondrial apoptotic pathway is regulated by various apoptosis-related genes, such as Bax and Bcl-xL [29]. Bax is a pro-apoptotic protein residing in the cytosol but translocates to the mitochondria upon the induction of apoptosis, whereas Bcl-xL is an anti-apoptotic protein that can inhibit Bax-induced apoptosis [30]. Western blot analysis revealed that the expression of Bax in the CCl_4_-treated model group was upregulated compared with that in the normal group, whereas the expression of Bcl-xL in the CCl_4_-treated model group was downregulated. However, the pre-administration of procyanidin B2 suppressed the upregulation of Bax expression and restored the downregulation of Bcl-xL expression induced by CCl_4_. These results indicated that procyanidin B2 may attenuate CCl_4_-induced hepatocyte apoptosis by regulating the expression of the apoptosis-related proteins Bax and Bcl-xL.

Many previous studies have proved that Grape seed extract (GSE), Grape seed procyanidin extract (GSPE), and proanthocyanidins showed protective effect against live-induced damage. Oral intake of GSE was found to attenuated histopathological changes in tamoxifen-induced liver injury, which restored the liver antioxidant enzymes levels and inhibited the elevated lipid peroxides as free radicals scavengers [31]. Pre-administration of GSE could reduce hepatic and damage induced by methotrexate-treatment in young rats as free radical scavenging [32]. In addition, GSE administered in a dose of 50 mg∙kg^−1^∙day^−1^ orally for 15 days before I/R injury and repeated before the reperfusion period was shown to reverse the levels of MDA, GSH and MPO activity induced by I/R. Serum AST and ALT levels as well as cytokines expression (TNF-α and IL-1β) were also down-regulated. Therefore, GSE reduced I/R-induced organ injury through its ability to balance the oxidant-antioxidant status, to inhibit neutrophil inﬁltration and to regulate the release of inﬂammatory mediators [33]. Moreover, in arsenic-induced liver injury, GSE co-treatment signiﬁcantly attenuated arsenic-induced low antioxidant defense, oxidative damage, proinﬂammatory cytokines and ﬁbrogenic genes through suppression of NADPH oxidase and TGF-β/Smad activation [34]. GSPE was also found to manifest effective hepatocellular protective action to ameliorate the developing liver fibrosis induced by chronic thioacetamide administration in mice. In this study, combined oral administration of GSPE at 100 mg∙kg^−1^ administration markedly suppressed lipid peroxidation, down-regulated the expression of the pro-inflammatory factors, including inducible iNOS and COX-2, and suppress the collagen accumulation [35]. Oral administration of proanthocyanidins (20 mg∙kg^−1^ daily for four weeks) also remarkably prevented the elevations in levels of serum alanine transaminase, aspartate transaminase, alkaline phosphatase, and bilirubin induced by dimethylnitrosamine in rats. It also restored serum albumin and reduced the hepatic level of malondialdehyde, which demonstrated that proanthocyanidins exhibited *in vivo* hepatoprotective and anti-fibrogenic effects against dimethylnitrosamine liver injury [36]. Although these extracts show powerful protective effect against induced liver injury, they are complex mixture of structurally related components. Therefore, the biological properties of individual components have not been explicitly determined. Meanwhile, there have been no studies on the protective effect of procyanidin B2 or extract containing procyanidin B2 against the CCl_4_-induced acute liver injury. Our work revealed that procyanidin B2 exhibited similar protective effects against induced acute hepatic injury as that in GSE or proanthocyanidins. Since Procyanidin B2 is one of the main components of GSPE with greater anti-inflammatory and antitumor effects than other components of GSPE, the further exploration in the preventive mechanisms of procyanidin B2 may supply a potential therapeutic agent for the induced hepatic injury.

The previous work has revealed that after procyanidin B2 administration, it is absorbed and excreted in urine, and a portion of the procyanidin B2 is degraded to (−)-epicatechin and to the metabolized conjugated and/or methylated (−)-epicatechin internally in the rat [37]. It also suggested that procyanidin B2 bioavailability was much lower than that of (−)-epicatechin in rat, which may be due to the differences in the solubility, lipophilicity, and excretion route (urinary or biliary) between procyanidin B2 and (−)-epicatechin. Our work has indicated that the protective effect is dose-dependent. Therefore, we are trying to investigate the suitable dose of procyanidin B2, which would keep its protective effect against induced liver injury as a nutritional supplement. Moreover, further studies are necessary to clarify the absorption mechanisms of procyanidin B2 and promote the elevation of procyanidin B2 bioavailability.

## 3. Experimental Section

### 3.1. Chemical and Reagents

Procyanidin B2, purchased from Chengdu Biopurify Phytochemicals Ltd. (Chengdu, China), was more than 95% pure by ultra-performance liquid chromatography (UPLC) analysis. The detection kits used for the determination of ALT activity, AST activity, SOD activity, CAT activity, GSH-Px activity and MDA contents were all purchased from Nanjing Jiancheng Institute of Biotechnology (Nanjing, China). The Nuclear/Cytoplasmic Protein Extraction kit, Tissue Protein Extraction Kit, and the Enhanced Bicinchoninic Acid (BCA) Protein Assay Kit were purchased from the Beyotime Institute of Biotechnology (Jiangsu, China). TRIzol reagent was purchased from Invitrogen (Carlsbad, CA, USA). The TaKaRa PrimeScript RT reagent kit was purchased from Takara Biotechnology Co., Ltd. (Dalian, China). The FastStart Universal SYBR Green Master (Rox) and *in Situ* Cell Death Detection Kit (TUNEL) were procured from Roche (Roche Diagnostic, Mannheim, Gemany). The primary antibodies against TNF-α, IL-1β, COX-2, iNOS, NF-κB p65, Bax, Bcl-xL, active caspase-3, β-actin, α-tubulin and lamin B1 were purchased from Cell Signaling Technology Inc. (Beverly, Amesbury, MA, USA). The secondary goat anti-mouse and goat anti-rabbit horseradish peroxidase (HRP)-conjugated antibodies were purchased from Santa Cruz Biotechnology (Santa Cruz, CA, USA). The enhanced chemiluminescence Western blot detection kit was obtained from Amersham. All other chemicals and reagents used were analytical grade.

### 3.2. Animals

Six-week-old male ICR mice were purchased from the Laboratory Animal Center, Yangzhou University (Yangzhou, China) and housed in environmentally controlled conditions (25 ± 2 °C and 12 h light: 12 h dark cycle, with the light cycle at 6:00 a.m.–6:00 p.m. and the dark cycle at 6:00 p.m.–6:00 a.m.) with *ad libitum* access to standard laboratory chow and water. The mice were acclimatized to laboratory condition for 7 days before the commencement of the experiment. The adaptation period and experiments were conducted in accordance with internationally accepted principles and national laws concerning the care and use of laboratory animals, with approved from the Ethical Committee of Nanjing University.

### 3.3. Experimental Design

After environmental adaptation, the mice were randomly allocated into six groups (*n* = 10). Group I (normal) was given distilled water by gavage for 7 days consecutively (10 mL∙kg^−1^ body weight). Group II (procyanidin B2 control) received procyanidin B2 by gavage for 7 days consecutively (100 mg∙kg^−1^, dissolved in water). Group III (model) was given distilled water by gavage for 7 days consecutively (10 mL∙kg^−1^ body weight). Groups IV, V, and VI were administered with procyanidin B2 by gavage (25, 50, and 100 mg∙kg^−1^, respectively, dissolved in water). After oral administration by gavage for 7 days consecutively, the mice in Groups III to VI were intraperitoneally injected with 0.3% (*v*/*v*) CCl_4_ (10 mL∙kg^−1^, dissolved in olive oil) on the eighth day, whereas the animals in Groups I and II intraperitoneally received equal volume of olive oil alone At 24 h after injection, the mice were sacrificed under ether anesthesia. Blood samples were collected, and serum was immediately separated. The liver tissue was isolated from each mouse and stored at −80 °C for further experiments.

### 3.4. Assessment of Liver Function

After blood collection, the serum was separated by centrifugation at 3000 rpm for 20 min at room temperature. Biochemical parameters of serum ALT and AST in mice were determined using the corresponding diagnostic kits in accordance with the manufacturer’s instructions.

### 3.5. Assay of Hepatic MDA, GSH-Px, SOD and CAT Levels

Each liver tissue sample was homogenized in nine volumes of ice cold 50 mM phosphate buffer (pH 7.4) and centrifuged at 2500 rpm for 20 min at 4 °C. Supernatant was used to determine the MDA, GSH-Px, SOD, CAT and total protein concentrations by using the commercially available diagnostic kits. The levels of MDA, GSH-Px, SOD, and CAT were normalized with the total protein content.

### 3.6. RNA Extraction and Quantitative Real Time PCR

Total RNA of the liver tissues was extracted using the TRIzol reagent. Reverse transcription was conducted with the TaKaRa PrimeScript RT reagent kit. The primers used in this study are listed in Table 1. For the internal standard control, the expression level of GAPDH was simultaneously quantified. Real-time PCR was performed with the SYBR Green PCR Kit and an ABI 7300 real-time PCR system for 40 cycles consisting of denaturation at 94 °C for 30 s, annealing at 60 °C for 30 s and extension at 72 °C for 30 s. All amplifications and detections were carried out in a MicroAmp optical 96-well reaction plate with optical adhesive covers (Applied Biosystems). Finally, the unknown amount of the template was calculated from the standard curve for quantitative analysis.

**Table 1:** PCR primers used in this study and the amplified product length.

Gene (Accession Number)	Primer Sequences (5′-3′)	Product Length (bp)
TNF-α (M11731)	Forward: GGCAGGTCTACTTTGGAGTC	233
	Reverse: CACTGTCCCAGCATCTTGTG	
IL-1β (NM_008361.3)	Forward: GCAGGCAGTATCACTCATTG	165
	Reverse: CACACCAGCAGGTTATCATC	
GAPDH (NM_008084.2)	Forward: CATCAACGGGAAGCCCATC	211
	Reverse: CTCGTGGTTCACACCCATC	

### 3.7. Histological Examination of Liver Tissue

For the histological investigations, liver tissues were removed from a portion of the left lobe, and cryosections were cut at 6 μm thickness. After hematoxylin and eosin (H & E) staining, the slides were observed for conventional morphological evaluation under a light microscope (Nikon Eclipse TE2000-U, NIKON, Tokyo, Japan) and photographed at 100× magnification.

### 3.8. TUNEL Assay

Mice liver cryosections were prepared for TUNEL assay, which was performed with a commercial kit in accordance with the manufacturer’s instructions. Briefly, the cryosections were fixed with 4% paraformaldehyde for 15 min at room temperature. The fixed sections were washed and incubated with proteinase K solution for 10 min. Subsequently, the FITC-labeled dUTP solution was added on the surface of the slides and incubated at 37 °C for 1 h. The labeled slices were washed and photographed under a fluorescence microscope (magnification, 100×). The TUNEL-positive cells that showed green florescence were calculated. Ten microscopic fields in each group were randomly selected to count the positive cells.

### 3.9. Western Blot Assay

At 24 h after CCl4 injection, the liver tissues were harvested and homogenized in RIPA lysis buffer (20 mM Tris PH 7.5, 150 mM NaCl, 1% Triton X-100, 2.5 mM sodium pyrophosphate, 1 mM EDTA, 1% Na_3_VO_4_, 0.5 μg∙mL^−1^ leupeptin, 1 mM phenylmethanesulfonyl fluoride (PMSF)) to prepare of whole protein extracts. The lysates were centrifuged at 12,000× *g* at 4 °C for 10 min. A nuclear/cytoplasmic protein extraction kit was used to extract nuclear proteins in accordance with the manufacturer’s instructions. Protein concentrations were determined with the BCA protein assay kit and 150 μg of protein were loaded per well on SDS-PAGE. Subsequently, proteins were transferred to PVDF membranes (Millipore, Billerica, MA, USA). Membranes were blocked at room temperature for 1 h with blocking solution (5% skimmed milk in Tris-buffered solution plus Tween-20 (TBST): 50 mM Tris-HCl, 150 mM NaCl, pH = 7.5, 0.1% *v*/*v* Tween 20). Membranes were then incubated overnight at 4 °C with primary antibodies for TNF-α, IL-1β, COX-2, iNOS, NF-κB p65, Bax, Bcl-xL, active caspase-3, β-actin, α-tubulin and lamin B1 in blocking solution. After three 10 min washing in TBST, membranes were incubated for 1 h at room temperature with a horseradish peroxidase-conjugated secondary antibody in blocking solution. The proteins were visualized using an enhanced chemiluminescence Western blot detection kit. The relative expression of target proteins was quantified by the Image-Analysis system. To eliminate the variations related to protein quantity and quality, the results were adjusted to β-actin, α-tubulin or lamin B1 expression.

### 3.10. Statistical Analysis

All data were reported as mean ± SE. Statistical analysis was conducted by one-way analysis of variance (ANOVA) followed by Scheffe’s *post hoc* test. A significant difference at *p* < 0.05 was accepted for all the tests.

## 4. Conclusions

In conclusion, the present study demonstrates the potent protective effects of procyanidin B2 against CCl_4_-induced acute liver damage. The hepatoprotective effects of procyanidin B2 depend on its ability to enhance the antioxidative defense system and downregulate the pro-inflammatory and apoptosis signal pathway. However, the exact molecular mechanism remains unclear and requires further investigation. Overall, our study provides evidence of the protective effects of procyanidin B2 on CCl_4_-induced liver damage and suggests procyanidin B2 as a potential hepatoprotective agent; its use in maintaining a healthy liver and preventing toxic liver damage deserves consideration and further examination.

## Abbreviations

ALT, alanine aminotransferase; AST, aspartate aminotransferase; GSPE, grape seed procyanidin extract; H & E, hematoxylin and eosin; SOD, superoxide dismutase; CAT, catalase; GSH-Px, glutathione peroxidase; MDA, malondialdehyde; ROS, reactive oxygen species; NF-κB, nuclear factor-kappa B; COX-2, cyclooxygenase-2; iNOS, inducible nitric oxide synthase; TUNEL, terminal-deoxynucleotidyl transferase mediated nick end labeling.
